# Insect Physical Control: Electric Field-Based Pest Management Approach

**DOI:** 10.3390/insects11080480

**Published:** 2020-07-29

**Authors:** Hideyoshi Toyoda

**Affiliations:** Research Association of Electric Field Screen Supporters, Nara 631-8505, Japan; toyoda@nara.kindai.ac.jp

**Keywords:** electric field screen, static electric field, dynamic electric field, attractive force, arc discharge exposure, practical implementation

## Abstract

The Special Issue ‘Insect physical control: electric field-based pest management approach’ was launched to showcase valuable new research on pest control using applied electrostatic engineering. Some phenomena generated in static and dynamic electric fields can be used to build new devices to capture or kill target insects using an attractive force or a force striking insects entering an electric field. This research field is new, and there are few researchers currently working within it. Consequently, this editorial introduces the history and general principles of electric field generation. I then discuss future directions for this field.

## 1. Generation and Characterization of Electric Fields

A conductor can be electrified by connecting it to a grounded negative- or positive-voltage generator. A voltage generator is used to increase the initial voltage to achieve the desired voltage using a transformer (coil) and a Cockcroft circuit integrated into an electric circuit in the voltage generator. The difference between negative- and positive-voltage generators is that the Cockcroft circuit is set so that negative electricity (i.e., free electrons) moves in the reverse direction. A negative-voltage generator draws free electrons from the ground (an infinite source or sink of electrons—in this case, a source) and supplies electrons to the conducting wire. A negative charge accumulates on the surface of the conductor. With an insulated conductor, negative charges on the conductor induce dielectric polarisation of the insulated coating of the conductor, negatively electrifying the outer surface of the insulator. A positive-voltage generator pushes free electrons to ground (an infinite sink of electrons) to positively charge the conductor (positive electrification). The surface of the conductor becomes positively charged due to electrostatic induction. In an insulated conductor, positive electrification of the conductor causes the insulating coating to become positively charged as a result of dielectric polarisation.

**Figure 1:**
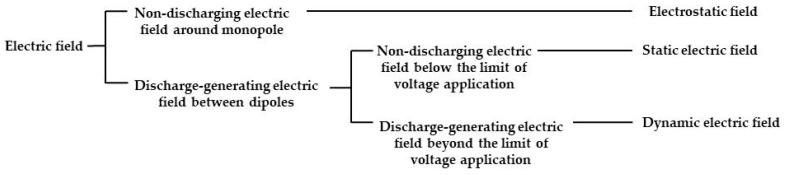
Classification of electric fields.

Figure 1 shows various electric-field configurations. An electrostatic field forms in the space surrounding a charged, insulated conductor used as a single pole. In this field, the electricity that accumulates on the charged conductor is not released (i.e., it is not discharged) in the field. By contrast, the electric field formed between oppositely charged poles may result in an electrical discharge if the applied voltage exceeds a particular limit, regardless of whether the conductor is insulated. This type of electric field is distinguished by the presence or absence of a discharge. Hereafter, a non-discharging electric field is referred to as a static electric field and a discharge-generating electric field as a dynamic electric field. The amount of electricity required for electrification is proportional to, and increases with, the applied voltage of the voltage generator. The applied voltage corresponds to the potential difference from the earth ground. In electrostatic and static electric fields, a larger potential difference creates more remarkable electrostatic phenomena, as a result of the greater attractive or repulsive force generated in the electric field. This allows the capture of spores and insect pests that enter the electric field. Especially, a static electric field exerts sufficient force to capture various insect pests. The main characteristic of a static electric field is the negative charge of the insulated conductor, which creates a strong repulsive force against other negative charges (electrons) in the electric field, pushing them toward the ground. Via this mechanism, any conductor that enters this field is deprived of its free electrons (negative electricity) and becomes positively charged. This phenomenon is called discharge-mediated positive electrification of the conductor. An insect that enters a static electric field is deprived of free electrons in the cuticle layer and becomes positively charged. Positively electrified insects are attracted to the insulated conductor. This force is so strong that the captured insect cannot escape. This capture mechanism is applicable to almost all insects.

A dynamic electric field causes the discharge of charged conductors. Factors related to the discharge of substances include the (1) voltage applied, (2) distance between poles, (3) potential difference between the two poles, (4) capacitance of the recipient receiving electricity, (5) air conductivity, and (6) volume resistivity of the insulator used for coverage. Of these factors, if an earthed conductor is one of the poles, discharge occurs more readily, as the earth receives electricity without any restriction. Assuming air is the medium, the volume of the conductive substances involved affects the insulation resistance of the air (i.e., air conductivity). A major factor is the vapour concentration in the air (i.e., the absolute humidity). The conductivity of the air increases with the vapour concentration, and an electric current is generated more easily in air under these conditions. Discharge is defined as electric current generation between opposite poles as a result of the dielectric breakdown of gases in the electric field by the potential difference between the poles. In a positively charged conductor, a corona discharge can change from a glow discharge (corona discharge surrounding the tip) to a streamer discharge via a brush-like discharge by increasing the applied voltage or decreasing the distance between the poles. The discharge finally breaks down into an arc discharge between the two poles. If the conductor is negatively charged, the corona is induced at lower voltages than at a positive needle pole. Although a glow corona with a short streamer discharge is generated, it does not grow and is followed by a complete breakdown in the form of an arc discharge. The strong force of a high-voltage-mediated arc discharge can dismember pests that enter an electric field.

## 2. Electric Field-Based Pest Management Approaches

The electric field screen, first introduced in 2006, is an air-shielding apparatus based on the principles and techniques of applied electrostatic engineering [1]. Initially, the apparatus was presented as a new device to capture the airborne conidia (spores) of phytopathogenic fungi during crop cultivation in a greenhouse. As the research progressed, the targets of the screen were expanded from fungal spores [2,3] to include flying insect pests [4,5], pollen grains that cause pollenosis [6], and fine particles of tobacco smoke [7,8] by optimising the structure of the electric field screen and its capture capabilities. Advances in electric field screen technology have allowed broader application of the device, from agricultural fields, e.g., crop production, processing, and storage, to environmental fields and public health science.

The two types of electric field screen used for insect capture were first reported in 2008 [4] and 2011 [5]. Subsequently, the focus of electric field screen research has been to (1) explain the mechanisms of insect capture [9,10,11,12], (2) devise practical applications to pest control [13,14,15,16,17], and (3) develop electric field screen devices for pest control [18,19,20,21,22]. These works provide an experimental basis for an alternative to conventional pest control. In two of the works mentioned above [5,14], we realised that insects find entering the static electric field of a single-charged dipolar electric field screen highly aversive. Avoidance behaviour has been detected in 82 insect species, belonging to 17 orders, 42 families, and 45 genera [23], which shed light on a new function of electric field screens; however, the question of how insects perceive an electric field remain unsolved and only one recent study has addressed this issue [24]. Additional studies are eagerly awaited.

Dynamic electric fields also have potential for novel physical measures to control insect pests. Two works [25,26] have reported unique apparatuses that cause an arc discharge to hit insects that enter a dynamic electric field. Arc-discharge-generating techniques may provide a new tool for detecting and dismembering insects nesting in dried grain or selectively killing flies emerging from underground pupae, which may be useful for organic farming. The corona discharge exposure technique has potential as a non-distractive inspection system to detect pests nesting in dried cereal products, based on the difference in conductivity between dried cereal grains and living pests. This is one of the main themes in this Special Issue.

## 3. Future Perspective

Future research may cover many topics. One is the installation of electric field screens on unmanned aerial vehicles for remote-controlled insect monitoring, especially flies transmitting human pathogens, like *Escherichia coli* O-157, over postharvest crops within facilities in the food supply chain. This requires replacing the heavy metal wires passed through an insulator tube with light materials to reduce the weight of the screen. Coating the inner surface of the insulator tube with a conductive paste is a potential alternative. The use of water in an insulative tube for the screen is another possible alternative, given its high conductivity. Interestingly, various water-soluble colourings can render the screen luminous, which may be useful for analysing insect photo-selectivity. A combination of insulated and uninsulated conductive fibres could be woven into the plastic sheet of an electric field screen. These are only several possible electric field-based pest control measures. Hopefully, this editorial leads to valuable research in this new field.
